# Bis(2,6-diamino­pyridinium) tartrate monohydrate

**DOI:** 10.1107/S1600536809044663

**Published:** 2009-10-31

**Authors:** Mohammad T. M. Al-Dajani, Hassan H. Abdallah, Nornisah Mohamed, Jia Hao Goh, Hoong-Kun Fun

**Affiliations:** a School of Pharmaceutical Sciences, Universiti Sains Malaysia, 11800 USM, Penang, Malaysia; b School of Chemical Sciences, Universiti Sains Malaysia, 11800 USM, Penang, Malaysia; c X-ray Crystallography Unit, School of Physics, Universiti Sains Malaysia, 11800 USM, Penang, Malaysia

## Abstract

In the title compound, 2C_5_H_8_N_3_^+^·C_4_H_4_O_6_^2−^·H_2_O, the two cations are essentially planar [maximum deviations = 0.023 (1) and 0.026 (1) Å]. In one of the cations, the protonated N atom and one of the amino group H atoms are hydrogen bonded to one of the carboxyl groups of the dianion through a pair of N—H⋯O hydrogen bonds, forming an *R*_2_^2^(8) ring motif. In the crystal structure, the tartrate anions and water mol­ecules are linked into chains along the *c* axis by inter­molecular O—H⋯O and C—H⋯O hydrogen bonds. The cations further link the anions and water mol­ecules into a three-dimensional extended structure by a network of N—H⋯O hydrogen bonds. The crystal structure is also stabilized by weak inter­molecular π–π inter­actions [centroid–centroid distance = 3.6950 (6) Å].

## Related literature

For related structures, see: Al-Dajani, Abdallah *et al.* (2009 ▶); Al-Dajani, Salhin *et al.* (2009 ▶). For hydrogen-bond motifs, see: Bernstein *et al.* (1995 ▶). For bond-length data, see: Allen *et al.* (1987 ▶). For the stability of the temperature controller used for the data collection, see: Cosier & Glazer (1986 ▶).
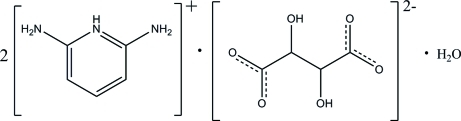


## Experimental

### 

#### Crystal data

2C_5_H_8_N_3_^+^·C_4_H_4_O_6_^2−^·H_2_O
*M* *_r_* = 386.38
Monoclinic, 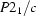

*a* = 14.4722 (2) Å
*b* = 15.7270 (2) Å
*c* = 7.8419 (1) Å
β = 96.916 (1)°
*V* = 1771.86 (4) Å^3^
*Z* = 4
Mo *K*α radiation
μ = 0.12 mm^−1^
*T* = 296 K
0.44 × 0.33 × 0.26 mm

#### Data collection

Bruker SMART APEXII CCD diffractometer
Absorption correction: multi-scan (**SADABS**; Bruker, 2005 ▶) *T*_min_ = 0.950, *T*_max_ = 0.970
55803 measured reflections
8384 independent reflections
5761 reflections with *I* > 2σ(*I*)
*R*_int_ = 0.029

#### Refinement

*R*[*F* ^2^ > 2σ(*F* ^2^)] = 0.048
*wR*(*F* ^2^) = 0.137
*S* = 1.04
8384 reflections
332 parameters
All H-atom parameters refined
Δρ_max_ = 0.33 e Å^−3^
Δρ_min_ = −0.24 e Å^−3^

### 

Data collection: *APEX2* (Bruker, 2005 ▶); cell refinement: *SAINT* (Bruker, 2005 ▶); data reduction: *SAINT*; program(s) used to solve structure: *SHELXTL* (Sheldrick, 2008 ▶); program(s) used to refine structure: *SHELXTL*; molecular graphics: *SHELXTL*; software used to prepare material for publication: *SHELXTL* and *PLATON* (Spek, 2009 ▶).

## Supplementary Material

Crystal structure: contains datablocks global, I. DOI: 10.1107/S1600536809044663/hb5185sup1.cif

Structure factors: contains datablocks I. DOI: 10.1107/S1600536809044663/hb5185Isup2.hkl

Additional supplementary materials:  crystallographic information; 3D view; checkCIF report

## Figures and Tables

**Table 1:** Hydrogen-bond geometry (Å, °)

*D*—H⋯*A*	*D*—H	H⋯*A*	*D*⋯*A*	*D*—H⋯*A*
O1—H1*O*1⋯O4^i^	0.900 (16)	1.840 (16)	2.7315 (10)	170.9 (15)
O2—H1*O*2⋯O4^i^	0.871 (17)	1.957 (17)	2.8260 (11)	175.6 (15)
N1—H1*N*1⋯O5	0.933 (13)	1.740 (13)	2.6660 (11)	171.4 (13)
N2—H1*N*2⋯O2^ii^	0.882 (17)	2.369 (17)	3.2254 (13)	163.7 (14)
N2—H1*N*2⋯O6^ii^	0.882 (17)	2.461 (17)	3.0531 (13)	125.0 (13)
N2—H2*N*2⋯O6	0.884 (14)	2.048 (14)	2.9306 (13)	176.7 (12)
N3—H1*N*3⋯O5	0.908 (16)	2.552 (16)	3.2431 (12)	133.4 (13)
N3—H1*N*3⋯O4^iii^	0.908 (16)	2.519 (17)	3.1842 (13)	130.5 (13)
N3—H2*N*3⋯O6^iv^	0.860 (16)	2.122 (16)	2.9499 (12)	161.3 (16)
N4—H1*N*4⋯O3^v^	0.924 (17)	1.810 (17)	2.7294 (12)	172.5 (15)
N5—H1*N*5⋯O1*W*^vi^	0.827 (19)	2.114 (19)	2.9265 (15)	167.7 (15)
N5—H2*N*5⋯O1^v^	0.888 (17)	2.243 (17)	3.0557 (14)	152.0 (14)
N5—H2*N*5⋯O3^v^	0.888 (17)	2.503 (16)	3.2154 (15)	137.8 (13)
N6—H1*N*6⋯O1*W*^vii^	0.901 (17)	2.127 (17)	3.0131 (18)	167.5 (14)
N6—H2*N*6⋯O1	0.87 (2)	2.189 (19)	2.9851 (15)	152 (2)
O1*W*—H1*W*1⋯O3^iii^	0.85 (2)	2.37 (3)	2.9371 (13)	125 (2)
O1*W*—H1*W*1⋯O4^iii^	0.85 (2)	2.42 (2)	3.1642 (13)	146 (2)
O1*W*—H2*W*1⋯O5	0.89 (2)	1.93 (2)	2.8153 (13)	175 (2)
C2—H2*A*⋯O3^iii^	0.963 (11)	2.490 (11)	3.3232 (12)	144.8 (9)

Symmetry codes: (i) 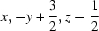; (ii) 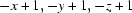; (iii) 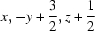; (iv) 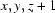; (v) 
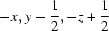
; (vi) 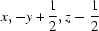; (vii) 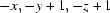.

**Fig. 1.**
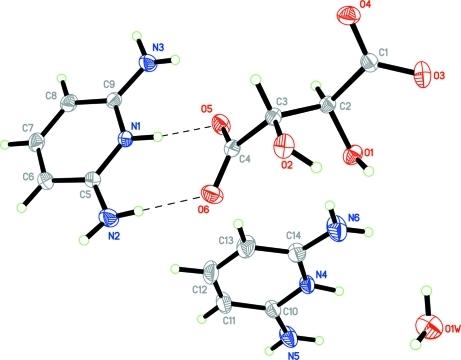
The molecular structure of (I), showing 30% probability displacement ellipsoids for non-H atoms. Intermolecular hydrogen bonds are shown as dashed lines.

**Fig. 2.**
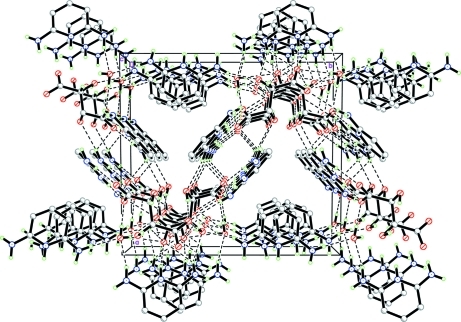
The crystal structure of (I), viewed along the c axis, showing the three-dimensional network. H atoms not involved in intermolecular interactions (dashed lines) have been omitted for clarity.

## supplementary crystallographic information

### Comment

The asymmetric unit of the title compound, (I) (Fig. 1), comprises of two 2,6-diaminopyridinium cations, a tartrate dianion and a water molecule. Two intermolecular protons transfer from the carboxylic groups of tartaric acid to atoms N1 & N4 of the 2,6-diaminopyridine moiety has resulted in the formation of ions. The protonated N1 atom and the N2 atom of the amino group are hydrogen bonded to the carboxyl group (atoms O5 & O6) *via* a pair of N—H···O hydrogen bonds forming an *R*^2^_2_(8) ring motif (Fig. 1, Bernstein *et al.*, 1995). The two 2,6-diaminopyridinium cations (N1–N3/C5–C9) and (N4–N6/C10–C14) are essentially planar, with maximum devations of 0.023 (1) and 0.026 (1) Å, respectively, for atoms C6 and C11. Comparing with the unprotonated structure (De cires-Mejias *et al.*, 2004), protonation of atoms N1 & N4 have widened the C—N—C angles to 123.51 (8) and 123.92 (9)°, respectively, for angles of C5—N1—C9 and C10—N4—C14. The bond lengths (Allen *et al.*, 1987) and angles observed are within normal ranges and are consistent with those related structures (Al-Dajani, Abdallah *et al.*, 2009; Al-Dajani, Salhin *et al.*, 2009).

The crystal structure of (I) is mainly stabilized by a network of N—H···O and C—H···O hydrogen bonds. Each N atom in the cations participates in intermolecular hydrogen bonds. In the crystal structure (Fig. 2), the tartrate anions and water molecules are linked into chains along the *c* axis by intermolecular O1—H1O1···O4, O2—H1O2···O4, O1W—H1W1···O3, O1W—H1W1···O4, O1W—H2W1···O5 and C2—H2A···O3 hydrogen bonds (Table 1). The 2,6-diaminopyridinium cations further linked the anions and water molecules into a three-dimensional extended structure by a network of N—H···O hydrogen bonds (Table 1). The crystal structure is further stabilized by weak intermolecular π–π interactions [*Cg*1···*Cg*1 = 3.6950 (6) Å; *Cg*1 is the centroid of the N1/C5–C9 pyridine ring].

### Experimental

Tartaric acid (0.01 mol, 1.5 g) was dissolved in 50 ml of methanol in a round bottom flask. 2,6-Diaminopyridine (0.02 mol, 2.2 g) was added in small portions to the flask with stirring. The reaction mixture was left stirring for 3 h at room temperature. Brown blocks of (I) were separated, washed with methanol and dried at 353 K.

### Refinement

All the hydrogen atoms were located from difference Fourier maps and allowed to refine freely [ranges: C—H = 0.922 (18) – 0.984 (15) Å; N—H = 0.827 (18) – 0.933 (14) Å]. The highest residual electron density peak is located at 0.77 Å from atom C2 and the deepest hole is located at 1.17 Å from atom C14.

### Crystal data

**Table:**

2C_5_H_8_N_3_^+^·C_4_H_4_O_6_^2^^−^·H_2_O	*F*(000) = 816
*M**_r_* = 386.38	*D*_x_ = 1.448 Mg m^−^^3^
Monoclinic, *P*2_1_/*c*	Mo *K*α radiation, λ = 0.71073 Å
Hall symbol: -P 2ybc	Cell parameters from 9979 reflections
*a* = 14.4722 (2) Å	θ = 2.6–33.1°
*b* = 15.7270 (2) Å	µ = 0.12 mm^−^^1^
*c* = 7.8419 (1) Å	*T* = 296 K
β = 96.916 (1)°	Block, brown
*V* = 1771.86 (4) Å^3^	0.44 × 0.33 × 0.26 mm
*Z* = 4	

### Data collection

**Table:**

Bruker SMART APEXII CCD diffractometer	8384 independent reflections
Radiation source: fine-focus sealed tube	5761 reflections with *I* > 2σ(*I*)
graphite	*R*_int_ = 0.029
φ and ω scans	θ_max_ = 36.1°, θ_min_ = 1.4°
Absorption correction: multi-scan (*SADABS*; Bruker, 2005)	*h* = −23→23
*T*_min_ = 0.950, *T*_max_ = 0.970	*k* = −25→24
55803 measured reflections	*l* = −12→12

### Refinement

**Table:**

Refinement on *F*^2^	Primary atom site location: structure-invariant direct methods
Least-squares matrix: full	Secondary atom site location: difference Fourier map
*R*[*F*^2^ > 2σ(*F*^2^)] = 0.048	Hydrogen site location: inferred from neighbouring sites
*wR*(*F*^2^) = 0.137	All H-atom parameters refined
*S* = 1.04	*w* = 1/[σ^2^(*F*_o_^2^) + (0.0645*P*)^2^ + 0.1774*P*] where *P* = (*F*_o_^2^ + 2*F*_c_^2^)/3
8384 reflections	(Δ/σ)_max_ < 0.001
332 parameters	Δρ_max_ = 0.33 e Å^−^^3^
0 restraints	Δρ_min_ = −0.23 e Å^−^^3^

### Special details

**Table:**

Geometry. All esds (except the esd in the dihedral angle between two l.s. planes) are estimated using the full covariance matrix. The cell esds are taken into account individually in the estimation of esds in distances, angles and torsion angles; correlations between esds in cell parameters are only used when they are defined by crystal symmetry. An approximate (isotropic) treatment of cell esds is used for estimating esds involving l.s. planes.
Refinement. Refinement of F^2^ against ALL reflections. The weighted R-factor wR and goodness of fit S are based on F^2^, conventional R-factors R are based on F, with F set to zero for negative F^2^. The threshold expression of F^2^ > 2sigma(F^2^) is used only for calculating R-factors(gt) etc. and is not relevant to the choice of reflections for refinement. R-factors based on F^2^ are statistically about twice as large as those based on F, and R- factors based on ALL data will be even larger.

### Fractional atomic coordinates and isotropic or equivalent isotropic displacement parameters (Å^2^)

**Table:**

	*x*	*y*	*z*	*U*_iso_*/*U*_eq_	
O1	0.14121 (5)	0.62179 (5)	0.37307 (9)	0.03748 (15)	
O2	0.33130 (5)	0.69021 (5)	0.36761 (10)	0.03834 (16)	
O3	0.09223 (6)	0.78743 (6)	0.35992 (11)	0.0505 (2)	
O4	0.19983 (6)	0.82535 (5)	0.57216 (9)	0.04158 (17)	
O5	0.28815 (5)	0.55133 (5)	0.71274 (9)	0.04001 (16)	
O6	0.36515 (6)	0.53101 (5)	0.48798 (9)	0.04409 (18)	
C1	0.15720 (6)	0.77090 (6)	0.47338 (11)	0.03144 (17)	
C2	0.18625 (6)	0.67770 (6)	0.49999 (10)	0.02824 (15)	
C3	0.29147 (6)	0.66622 (6)	0.51725 (10)	0.02834 (15)	
C4	0.31696 (6)	0.57521 (6)	0.57453 (10)	0.02985 (16)	
N1	0.39658 (5)	0.44405 (5)	0.90954 (9)	0.02900 (14)	
N2	0.47289 (7)	0.39813 (7)	0.68421 (11)	0.0423 (2)	
N3	0.31436 (7)	0.50158 (7)	1.11702 (12)	0.0429 (2)	
C5	0.46341 (6)	0.39419 (6)	0.85246 (11)	0.03136 (17)	
C6	0.51647 (7)	0.34231 (7)	0.97048 (14)	0.0404 (2)	
C7	0.49815 (8)	0.34277 (7)	1.13904 (14)	0.0427 (2)	
C8	0.43089 (8)	0.39418 (7)	1.19503 (12)	0.0395 (2)	
C9	0.38033 (6)	0.44752 (6)	1.07627 (10)	0.03057 (16)	
N4	0.07411 (6)	0.29439 (6)	0.34459 (12)	0.03809 (18)	
N5	0.05279 (8)	0.15005 (7)	0.31936 (18)	0.0562 (3)	
N6	0.07972 (10)	0.44052 (8)	0.3524 (2)	0.0649 (3)	
C10	0.10707 (7)	0.21441 (7)	0.37940 (14)	0.0394 (2)	
C11	0.19414 (8)	0.20559 (8)	0.47490 (18)	0.0504 (3)	
C12	0.24392 (8)	0.27782 (10)	0.52373 (19)	0.0564 (3)	
C13	0.20988 (9)	0.35831 (9)	0.48473 (18)	0.0534 (3)	
C14	0.12190 (8)	0.36637 (7)	0.39441 (15)	0.0426 (2)	
O1W	0.11303 (7)	0.52769 (6)	0.82780 (15)	0.0559 (2)	
H1O1	0.1548 (11)	0.6424 (10)	0.272 (2)	0.057 (4)*	
H1O2	0.2904 (13)	0.6826 (10)	0.278 (2)	0.067 (5)*	
H1N1	0.3619 (9)	0.4801 (8)	0.8319 (18)	0.044 (3)*	
H1N2	0.5253 (12)	0.3800 (10)	0.650 (2)	0.062 (4)*	
H2N2	0.4425 (9)	0.4390 (9)	0.6238 (18)	0.045 (3)*	
H1N3	0.2911 (11)	0.5406 (11)	1.038 (2)	0.065 (5)*	
H2N3	0.3161 (12)	0.5144 (10)	1.224 (2)	0.067 (5)*	
H1N4	0.0176 (12)	0.2971 (10)	0.276 (2)	0.059 (4)*	
H1N5	0.0716 (12)	0.1009 (12)	0.338 (2)	0.068 (5)*	
H2N5	−0.0001 (12)	0.1605 (10)	0.253 (2)	0.059 (4)*	
H1N6	0.0204 (12)	0.4416 (10)	0.302 (2)	0.060 (4)*	
H2N6	0.1102 (14)	0.4860 (12)	0.389 (3)	0.079 (5)*	
H2A	0.1657 (8)	0.6614 (7)	0.6076 (14)	0.030 (3)*	
H3A	0.3165 (9)	0.7015 (7)	0.6120 (16)	0.034 (3)*	
H6A	0.5634 (12)	0.3070 (10)	0.937 (2)	0.063 (4)*	
H7A	0.5370 (10)	0.3053 (9)	1.2187 (18)	0.050 (4)*	
H8A	0.4196 (10)	0.3943 (9)	1.3120 (19)	0.051 (4)*	
H11A	0.2168 (12)	0.1515 (11)	0.498 (2)	0.067 (5)*	
H12A	0.3045 (13)	0.2700 (11)	0.588 (2)	0.075 (5)*	
H13A	0.2436 (12)	0.4097 (11)	0.514 (2)	0.069 (5)*	
H1W1	0.1128 (16)	0.5645 (16)	0.907 (3)	0.102 (7)*	
H2W1	0.1669 (15)	0.5349 (13)	0.785 (3)	0.087 (6)*	

### Atomic displacement parameters (Å^2^)

**Table:**

	*U*^11^	*U*^22^	*U*^33^	*U*^12^	*U*^13^	*U*^23^
O1	0.0370 (3)	0.0357 (4)	0.0381 (3)	−0.0092 (3)	−0.0024 (3)	−0.0016 (3)
O2	0.0333 (3)	0.0438 (4)	0.0384 (3)	−0.0046 (3)	0.0061 (3)	0.0108 (3)
O3	0.0395 (4)	0.0544 (5)	0.0547 (5)	0.0125 (3)	−0.0065 (3)	0.0098 (4)
O4	0.0576 (4)	0.0305 (3)	0.0367 (3)	0.0000 (3)	0.0057 (3)	−0.0021 (3)
O5	0.0451 (4)	0.0428 (4)	0.0335 (3)	0.0128 (3)	0.0100 (3)	0.0117 (3)
O6	0.0569 (4)	0.0413 (4)	0.0360 (3)	0.0153 (3)	0.0133 (3)	0.0013 (3)
C1	0.0326 (4)	0.0333 (4)	0.0292 (3)	0.0048 (3)	0.0066 (3)	0.0038 (3)
C2	0.0298 (4)	0.0285 (4)	0.0261 (3)	−0.0009 (3)	0.0020 (3)	0.0011 (3)
C3	0.0292 (4)	0.0281 (4)	0.0269 (3)	−0.0014 (3)	−0.0002 (3)	0.0004 (3)
C4	0.0315 (4)	0.0315 (4)	0.0257 (3)	0.0023 (3)	−0.0001 (3)	0.0008 (3)
N1	0.0305 (3)	0.0292 (4)	0.0266 (3)	0.0036 (3)	0.0006 (2)	0.0020 (2)
N2	0.0456 (5)	0.0480 (5)	0.0346 (4)	0.0104 (4)	0.0100 (3)	0.0000 (4)
N3	0.0471 (5)	0.0488 (5)	0.0331 (4)	0.0141 (4)	0.0064 (3)	−0.0019 (4)
C5	0.0311 (4)	0.0300 (4)	0.0325 (4)	0.0013 (3)	0.0018 (3)	−0.0028 (3)
C6	0.0396 (5)	0.0367 (5)	0.0431 (5)	0.0124 (4)	−0.0028 (4)	−0.0020 (4)
C7	0.0485 (5)	0.0359 (5)	0.0398 (5)	0.0078 (4)	−0.0106 (4)	0.0039 (4)
C8	0.0483 (5)	0.0411 (5)	0.0274 (4)	0.0037 (4)	−0.0022 (3)	0.0033 (3)
C9	0.0322 (4)	0.0317 (4)	0.0270 (3)	−0.0004 (3)	0.0004 (3)	−0.0007 (3)
N4	0.0290 (4)	0.0349 (4)	0.0485 (4)	0.0013 (3)	−0.0029 (3)	−0.0015 (3)
N5	0.0403 (5)	0.0351 (5)	0.0901 (9)	0.0016 (4)	−0.0046 (5)	−0.0084 (5)
N6	0.0575 (7)	0.0332 (5)	0.0982 (10)	−0.0015 (5)	−0.0145 (6)	0.0028 (5)
C10	0.0319 (4)	0.0352 (5)	0.0508 (5)	0.0024 (4)	0.0031 (4)	−0.0024 (4)
C11	0.0376 (5)	0.0459 (7)	0.0651 (7)	0.0093 (5)	−0.0048 (5)	0.0021 (5)
C12	0.0339 (5)	0.0639 (8)	0.0670 (8)	0.0027 (5)	−0.0123 (5)	−0.0004 (6)
C13	0.0413 (6)	0.0490 (7)	0.0661 (8)	−0.0081 (5)	−0.0095 (5)	−0.0043 (5)
C14	0.0388 (5)	0.0365 (5)	0.0512 (6)	−0.0026 (4)	−0.0003 (4)	−0.0007 (4)
O1W	0.0539 (5)	0.0414 (5)	0.0734 (6)	−0.0122 (4)	0.0114 (4)	−0.0059 (4)

### Geometric parameters (Å, °)

**Table:**

O1—C2	1.4257 (11)	C6—H6A	0.938 (17)
O1—H1O1	0.901 (16)	C7—C8	1.3773 (16)
O2—C3	1.4194 (10)	C7—H7A	0.984 (15)
O2—H1O2	0.868 (19)	C8—C9	1.3937 (13)
O3—C1	1.2418 (12)	C8—H8A	0.951 (14)
O4—C1	1.2644 (12)	N4—C14	1.3591 (14)
O5—C4	1.2643 (10)	N4—C10	1.3613 (13)
O6—C4	1.2426 (11)	N4—H1N4	0.923 (17)
C1—C2	1.5322 (13)	N5—C10	1.3319 (15)
C2—C3	1.5231 (12)	N5—H1N5	0.827 (18)
C2—H2A	0.962 (11)	N5—H2N5	0.888 (17)
C3—C4	1.5318 (12)	N6—C14	1.3390 (16)
C3—H3A	0.962 (12)	N6—H1N6	0.901 (18)
N1—C9	1.3572 (11)	N6—H2N6	0.87 (2)
N1—C5	1.3620 (11)	C10—C11	1.3930 (16)
N1—H1N1	0.933 (14)	C11—C12	1.3747 (19)
N2—C5	1.3443 (12)	C11—H11A	0.922 (18)
N2—H1N2	0.881 (17)	C12—C13	1.379 (2)
N2—H2N2	0.884 (14)	C12—H12A	0.963 (19)
N3—C9	1.3453 (13)	C13—C14	1.3862 (16)
N3—H1N3	0.907 (17)	C13—H13A	0.959 (17)
N3—H2N3	0.859 (18)	O1W—H1W1	0.85 (3)
C5—C6	1.3931 (13)	O1W—H2W1	0.89 (2)
C6—C7	1.3790 (15)		
			
C2—O1—H1O1	105.3 (10)	C8—C7—C6	122.24 (9)
C3—O2—H1O2	108.9 (11)	C8—C7—H7A	121.3 (8)
O3—C1—O4	124.66 (9)	C6—C7—H7A	116.4 (8)
O3—C1—C2	118.00 (9)	C7—C8—C9	118.34 (9)
O4—C1—C2	117.31 (8)	C7—C8—H8A	121.2 (9)
O1—C2—C3	110.92 (7)	C9—C8—H8A	120.4 (9)
O1—C2—C1	113.58 (7)	N3—C9—N1	117.68 (8)
C3—C2—C1	112.37 (7)	N3—C9—C8	123.52 (9)
O1—C2—H2A	106.5 (7)	N1—C9—C8	118.79 (8)
C3—C2—H2A	107.6 (7)	C14—N4—C10	123.92 (9)
C1—C2—H2A	105.4 (7)	C14—N4—H1N4	120.8 (10)
O2—C3—C2	113.34 (7)	C10—N4—H1N4	115.1 (10)
O2—C3—C4	112.54 (7)	C10—N5—H1N5	118.6 (12)
C2—C3—C4	109.86 (7)	C10—N5—H2N5	119.8 (11)
O2—C3—H3A	109.4 (7)	H1N5—N5—H2N5	121.5 (16)
C2—C3—H3A	106.2 (7)	C14—N6—H1N6	120.4 (11)
C4—C3—H3A	104.9 (7)	C14—N6—H2N6	115.8 (13)
O6—C4—O5	124.53 (9)	H1N6—N6—H2N6	123.2 (17)
O6—C4—C3	119.58 (8)	N5—C10—N4	116.98 (10)
O5—C4—C3	115.86 (8)	N5—C10—C11	124.84 (11)
C9—N1—C5	123.51 (8)	N4—C10—C11	118.18 (10)
C9—N1—H1N1	117.5 (8)	C12—C11—C10	118.51 (11)
C5—N1—H1N1	118.9 (8)	C12—C11—H11A	123.0 (11)
C5—N2—H1N2	118.3 (11)	C10—C11—H11A	118.4 (11)
C5—N2—H2N2	117.2 (9)	C11—C12—C13	122.37 (11)
H1N2—N2—H2N2	117.8 (13)	C11—C12—H12A	116.9 (11)
C9—N3—H1N3	118.8 (10)	C13—C12—H12A	120.7 (11)
C9—N3—H2N3	116.2 (11)	C12—C13—C14	118.61 (11)
H1N3—N3—H2N3	118.1 (15)	C12—C13—H13A	124.2 (10)
N2—C5—N1	117.16 (8)	C14—C13—H13A	117.1 (10)
N2—C5—C6	124.46 (9)	N6—C14—N4	116.97 (10)
N1—C5—C6	118.38 (8)	N6—C14—C13	124.67 (11)
C7—C6—C5	118.65 (9)	N4—C14—C13	118.36 (11)
C7—C6—H6A	119.9 (10)	H1W1—O1W—H2W1	106 (2)
C5—C6—H6A	121.5 (10)		
			
O3—C1—C2—O1	7.58 (11)	C5—C6—C7—C8	−1.59 (17)
O4—C1—C2—O1	−174.21 (7)	C6—C7—C8—C9	−0.26 (17)
O3—C1—C2—C3	134.52 (9)	C5—N1—C9—N3	177.76 (9)
O4—C1—C2—C3	−47.27 (10)	C5—N1—C9—C8	−3.54 (14)
O1—C2—C3—O2	65.36 (10)	C7—C8—C9—N3	−178.63 (10)
C1—C2—C3—O2	−62.99 (9)	C7—C8—C9—N1	2.75 (15)
O1—C2—C3—C4	−61.50 (9)	C14—N4—C10—N5	−178.83 (11)
C1—C2—C3—C4	170.15 (7)	C14—N4—C10—C11	1.42 (17)
O2—C3—C4—O6	−2.16 (12)	N5—C10—C11—C12	177.88 (13)
C2—C3—C4—O6	125.14 (9)	N4—C10—C11—C12	−2.39 (19)
O2—C3—C4—O5	176.12 (8)	C10—C11—C12—C13	1.4 (2)
C2—C3—C4—O5	−56.57 (10)	C11—C12—C13—C14	0.6 (2)
C9—N1—C5—N2	−178.91 (9)	C10—N4—C14—N6	−179.17 (12)
C9—N1—C5—C6	1.66 (14)	C10—N4—C14—C13	0.63 (17)
N2—C5—C6—C7	−178.45 (10)	C12—C13—C14—N6	178.15 (14)
N1—C5—C6—C7	0.93 (15)	C12—C13—C14—N4	−1.64 (19)

### Hydrogen-bond geometry (Å, °)

**Table:**

*D*—H···*A*	*D*—H	H···*A*	*D*···*A*	*D*—H···*A*
O1—H1O1···O4^i^	0.900 (16)	1.840 (16)	2.7315 (10)	170.9 (15)
O2—H1O2···O4^i^	0.871 (17)	1.957 (17)	2.8260 (11)	175.6 (15)
N1—H1N1···O5	0.933 (13)	1.740 (13)	2.6660 (11)	171.4 (13)
N2—H1N2···O2^ii^	0.882 (17)	2.369 (17)	3.2254 (13)	163.7 (14)
N2—H1N2···O6^ii^	0.882 (17)	2.461 (17)	3.0531 (13)	125.0 (13)
N2—H2N2···O6	0.884 (14)	2.048 (14)	2.9306 (13)	176.7 (12)
N3—H1N3···O5	0.908 (16)	2.552 (16)	3.2431 (12)	133.4 (13)
N3—H1N3···O4^iii^	0.908 (16)	2.519 (17)	3.1842 (13)	130.5 (13)
N3—H2N3···O6^iv^	0.860 (16)	2.122 (16)	2.9499 (12)	161.3 (16)
N4—H1N4···O3^v^	0.924 (17)	1.810 (17)	2.7294 (12)	172.5 (15)
N5—H1N5···O1W^vi^	0.827 (19)	2.114 (19)	2.9265 (15)	167.7 (15)
N5—H2N5···O1^v^	0.888 (17)	2.243 (17)	3.0557 (14)	152.0 (14)
N5—H2N5···O3^v^	0.888 (17)	2.503 (16)	3.2154 (15)	137.8 (13)
N6—H1N6···O1W^vii^	0.901 (17)	2.127 (17)	3.0131 (18)	167.5 (14)
N6—H2N6···O1	0.87 (2)	2.189 (19)	2.9851 (15)	152 (2)
O1W—H1W1···O3^iii^	0.85 (2)	2.37 (3)	2.9371 (13)	125 (2)
O1W—H1W1···O4^iii^	0.85 (2)	2.42 (2)	3.1642 (13)	146 (2)
O1W—H2W1···O5	0.89 (2)	1.93 (2)	2.8153 (13)	175 (2)
C2—H2A···O3^iii^	0.963 (11)	2.490 (11)	3.3232 (12)	144.8 (9)

Symmetry codes: (i) *x*, −*y*+3/2, *z*−1/2; (ii) −*x*+1, −*y*+1, −*z*+1; (iii) *x*, −*y*+3/2, *z*+1/2; (iv) *x*, *y*, *z*+1; (v) −*x*, *y*−1/2, −*z*+1/2; (vi) *x*, −*y*+1/2, *z*−1/2; (vii) −*x*, −*y*+1, −*z*+1.
